# Bis(2,6-di­methyl­anilinium) di­aqua­bis­(di­hydrogen diphosphato-κ^2^ *O*,*O*′)cobaltate(II)

**DOI:** 10.1107/S1600536814002530

**Published:** 2014-02-08

**Authors:** Ahlem Ben Saad, Ahmed Selmi, Mohamed Rzaigui, Samah Toumi Akriche

**Affiliations:** a Laboratoire de Chimie des Matériaux, Faculté des Sciences de Bizerte, 7021 Zarzouna Bizerte, Tunisia

## Abstract

In the title compound, (C_8_H_12_N)_2_[Co(H_2_P_2_O_7_)_2_(H_2_O)_2_], the Co^2+^ ion lies on a crystallographic inversion centre and adopts a slightly distorted octa­hedral CoO_6_ coordination geometry arising from two chelating diphosphate [H_2_P_2_O_7_]^2−^ ligands and two *trans* water mol­ecules. In the crystal, the components are linked by O—H⋯O, N—H⋯O and C—H⋯O hydrogen bonds and weak aromatic π–π stacking [shortest centroid–centroid separation = 3.778 (2) Å] inter­actions. (001) layers of alternating organic cations and complex inorganic anions are apparent.

## Related literature   

For related structures, see: Ahmed *et al.* (2006 ▶); Selmi *et al.* (2006 ▶, 2009 ▶); Gharbi *et al.* (1994 ▶); Gharbi & Jouini (2004 ▶); Elboulali *et al.* (2013*a* ▶,*b* ▶); Essehli *et al.* (2006 ▶).
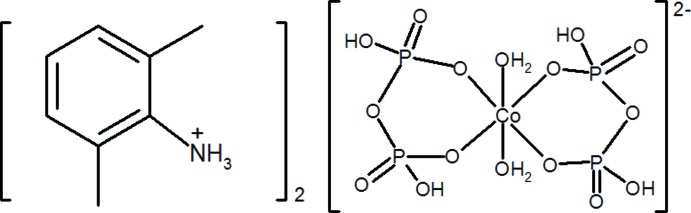


## Experimental   

### 

#### Crystal data  

(C_8_H_12_N)_2_[Co(H_2_P_2_O_7_)_2_(H_2_O)_2_]
*M* *_r_* = 691.25
Triclinic, 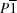

*a* = 7.320 (3) Å
*b* = 7.584 (4) Å
*c* = 13.413 (2) Å
α = 85.35 (3)°
β = 75.56 (2)°
γ = 74.42 (5)°
*V* = 694.5 (5) Å^3^
*Z* = 1
Ag *K*α radiation
λ = 0.56087 Å
μ = 0.48 mm^−1^
*T* = 293 K
0.40 × 0.30 × 0.20 mm

#### Data collection  

Enraf–Nonius CAD-4 diffractometer
Absorption correction: ψ scan (North *et al.*, 1968 ▶) *T*_min_ = 0.799, *T*_max_ = 0.982
9085 measured reflections
6683 independent reflections
5514 reflections with *I* > 2σ(*I*)
*R*_int_ = 0.080
2 standard reflections every 120 min intensity decay: 5%

#### Refinement  

*R*[*F* ^2^ > 2σ(*F* ^2^)] = 0.061
*wR*(*F* ^2^) = 0.168
*S* = 1.04
6683 reflections
189 parameters
3 restraints
H atoms treated by a mixture of independent and constrained refinement
Δρ_max_ = 1.57 e Å^−3^
Δρ_min_ = −0.82 e Å^−3^

### 

Data collection: *CAD-4 EXPRESS* (Enraf–Nonius, 1994 ▶); cell refinement: *CAD-4 EXPRESS*; data reduction: *XCAD4* (Harms & Wocadlo, 1996 ▶); program(s) used to solve structure: *SHELXS97* (Sheldrick, 2008 ▶); program(s) used to refine structure: *SHELXL97* (Sheldrick, 2008 ▶); molecular graphics: *ORTEP-3 for Windows* (Farrugia, 2012 ▶) and *DIAMOND* (Brandenburg & Putz, 2005 ▶); software used to prepare material for publication: *WinGX* (Farrugia, 2012 ▶).

## Supplementary Material

Crystal structure: contains datablock(s) I. DOI: 10.1107/S1600536814002530/hb7194sup1.cif

Structure factors: contains datablock(s) I. DOI: 10.1107/S1600536814002530/hb7194Isup2.hkl

CCDC reference:

Additional supporting information:  crystallographic information; 3D view; checkCIF report

## Figures and Tables

**Table 1:** Selected bond lengths (Å)

Co1—O5	2.0645 (18)
Co1—O1	2.0744 (17)
Co1—O1*W*	2.130 (2)

**Table 2:** Hydrogen-bond geometry (Å, °)

*D*—H⋯*A*	*D*—H	H⋯*A*	*D*⋯*A*	*D*—H⋯*A*
O2—H2⋯O6^i^	0.82	1.72	2.532 (3)	174
O7—H7⋯O3^ii^	0.82	1.70	2.505 (3)	167
O1*W*—H2*W*1⋯O4^i^	0.87 (2)	2.11 (2)	2.947 (3)	161 (4)
O1*W*—H1*W*1⋯O7^iii^	0.86 (2)	1.96 (2)	2.813 (3)	177 (4)
N1—H1*C*⋯O6^iv^	0.89	1.94	2.828 (3)	175
N1—H1*A*⋯O3^ii^	0.89	1.93	2.805 (3)	168
N1—H1*B*⋯O5	0.89	2.29	3.005 (3)	138
N1—H1*B*⋯O1^v^	0.89	2.37	3.016 (3)	129
C7—H7*C*⋯O2^ii^	0.96	2.58	3.497 (5)	160
C7—H7*A*⋯O6^iv^	0.96	2.57	3.343 (4)	138

Symmetry codes: (i) 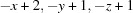; (ii) 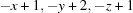; (iii) 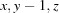; (iv) 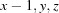; (v) 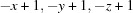.

**Fig. 1.**
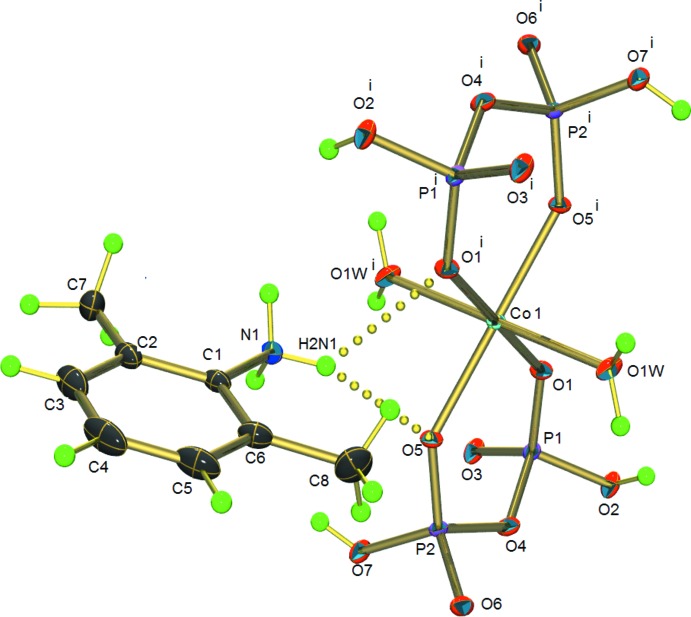
An ORTEP view of (I) with displacement ellipsoids drawn at the 30% probability level. H atoms are represented as small spheres of arbitrary radii. Hydrogen bonds are represented as dashed lines. [Symmetry code: (i) 1 - x, 1 - y, 1 - z.]

**Fig. 2.**
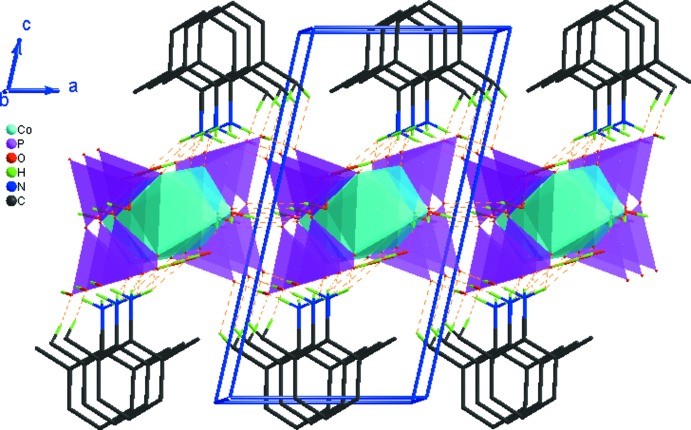
Perspetive view of crystal packing of (I). The H-atoms not involved in H-bonding are omitted.

## supplementary crystallographic information

### 1. Comment

The present work is a part of our investigation of diphosphate materials of mixed organic-metal cations. Here, we report a new synthesized one: (C_8_H_12_N)_2_[Co(H_2_P_2_O_7_)_2_(H_2_O)_2_] (I).

The asymmetric unit of (I) is made up of a half of mononuclear [Co(H_2_P_2_O_7_)_2_(H_2_O)_2_]^2-^ moiety and one of *2*,*6*-xylidinium cation. As Co^2+^ ion lie on inversion centre, the complete formula unit is generated by this element of symmetry (Fig. 1).

The inorganic component is made up of anionic monomeric units of formula [Co(H_2_P_2_O_7_)_2_(H_2_O)_2_]^2-^, in which the six-coordinated cobalt (II) ions are linked by means of O—H···O (with 2.505 (3) Å < O···O < 2.947 (3) Å see Table 1) hydrogen bonds to develop anionic layers running perpendicular to *c* axis. Similar [*M*(H_2_P_2_O_7_)_2_(H_2_O)_2_]^2n-^ two-dimensional topological framework can be also found in the previously reported diphosphate materials (Selmi *et al.*, 2006, 2009; Elboulali *et al.*, 2013*a*,*b*). The cobalt (II) complex adopts a distorted octahedral where the four O atoms of diphospahte bidentate ligand (O1, O5, O1^i^ and O5^i^ with (i); -*x* + 1, -*y* + 1, -*z* + 1) fill the equatorial positions and the two symmetry equivalent O atoms of water molecules (O1W, O1W^i^ with (i); -*x* + 1, -*y* + 1, -*z* + 1) occupy the apical ones. The bond distances and angles in CoO_6_ are sufficiently close to those found in the related *M*^II^O_6_ complexes featuring the chelating diphosphate ligand (Ahmed *et al.*, 2006; Elboulali *et al.*, 2013*a*, 2013*b*; Essehli *et al.*, 2006; Gharbi *et al.*, 1994; Gharbi *et al.*, 2004; Selmi *et al.*, 2006 and 2009).

Two phosphorous atoms are tetrahedrally coordinated and covalently linked through O4 to form a P_2_O_7_ group with bent geometry (P1–O4–P2 = 129.66 (12) °) and quasi-eclipsed conformation as confirmed by the torsion angle values 11.66°, 5.76° and 7.16° respectively of O5—P2—P1—O1, O7—P2—P1—O3 and O6—P2—P1—O2.

In the crystal of 1, the 2,6-xylidinium cations are linked by means N—H···O and C—H···O hydrogen bonds to inorganic layers thanks to NH_3_ group of the protonated cations (Fig. 2). It's to be noted that two adjacent cations interact by weak π···π stacking interactions (values of the inter-planar distances of 3.778 (2) Å).

### 2. Experimental

Pink blocks of the title compound were grown at room temperature by slow evaporation from water-ethanol (80/20) solution containing a stoichiometric mixture of CoCl_2_·6H_2_O (0.12 mg, 0.5 mmol), *2*,*6*-xylidine (0.12 mL,1 mmol) and N_4_P_2_O_7_.10H_2_O (0.45 mg, 1 mmol) dissolved in 2 ml of hydrochloric acid solution (2*M*).

### 3. Refinement

Due to poor quality of the crystal when being diffracted, some bad reflections (with (Iobs-Icalc)/SigmaW > 10) are observed. For the final refinement they are omitted. H atoms attached to C, O and N atoms were fixed geometrically and treated as riding, with C—H = 0.93 Å with *U*_iso_(H) = 1.2Ueq(C) for aromatic ring and C—H = 0.96 Å and N—H = 0.89 Å respectively for CH_3_ and NH_3_ cation radicals and O—H = 0.82 Å for diphosphoric anion with *U*_iso_(H) = 1.5Ueq(C, O or N). The water H atoms were refined using restraints [O—H = 0.85 (1) A °, H···H = 1.44 (2) A ° and *U*_iso_(H) = 1.5Ueq(O)].

### Crystal data

**Table:**

(C_8_H_12_N)_2_[Co(H_2_P_2_O_7_)_2_(H_2_O)_2_]	*Z* = 1
*M**_r_* = 691.25	*F*(000) = 357
Triclinic, *P*1	*D*_x_ = 1.653 Mg m^−^^3^
*a* = 7.320 (3) Å	Ag *K*α radiation, λ = 0.56087 Å
*b* = 7.584 (4) Å	Cell parameters from 25 reflections
*c* = 13.413 (2) Å	θ = 9–11°
α = 85.35 (3)°	µ = 0.48 mm^−^^1^
β = 75.56 (2)°	*T* = 293 K
γ = 74.42 (5)°	Prism, pink
*V* = 694.5 (5) Å^3^	0.40 × 0.30 × 0.20 mm

### Data collection

**Table:**

Enraf–Nonius CAD-4 diffractometer	5514 reflections with *I* > 2σ(*I*)
Radiation source: fine-focus sealed tube	*R*_int_ = 0.080
Graphite monochromator	θ_max_ = 28.0°, θ_min_ = 2.2°
non–profiled ω scans	*h* = −12→12
Absorption correction: ψ scan (North *et al.*, 1968)	*k* = −12→12
*T*_min_ = 0.799, *T*_max_ = 0.982	*l* = −5→22
9085 measured reflections	2 standard reflections every 120 min
6683 independent reflections	intensity decay: 5%

### Refinement

**Table:**

Refinement on *F*^2^	Primary atom site location: structure-invariant direct methods
Least-squares matrix: full	Secondary atom site location: difference Fourier map
*R*[*F*^2^ > 2σ(*F*^2^)] = 0.061	Hydrogen site location: inferred from neighbouring sites
*wR*(*F*^2^) = 0.168	H atoms treated by a mixture of independent and constrained refinement
*S* = 1.04	*w* = 1/[σ^2^(*F*_o_^2^) + (0.0826*P*)^2^ + 0.9452*P*] where *P* = (*F*_o_^2^ + 2*F*_c_^2^)/3
6683 reflections	(Δ/σ)_max_ = 0.005
189 parameters	Δρ_max_ = 1.57 e Å^−^^3^
3 restraints	Δρ_min_ = −0.82 e Å^−^^3^

### Special details

**Table:**

Geometry. All e.s.d.'s (except the e.s.d. in the dihedral angle between two l.s. planes) are estimated using the full covariance matrix. The cell e.s.d.'s are taken into account individually in the estimation of e.s.d.'s in distances, angles and torsion angles; correlations between e.s.d.'s in cell parameters are only used when they are defined by crystal symmetry. An approximate (isotropic) treatment of cell e.s.d.'s is used for estimating e.s.d.'s involving l.s. planes.
Refinement. Refinement of *F*^2^ against ALL reflections. The weighted *R*-factor *wR* and goodness of fit *S* are based on *F*^2^, conventional *R*-factors *R* are based on *F*, with *F* set to zero for negative *F*^2^. The threshold expression of *F*^2^ > σ(*F*^2^) is used only for calculating *R*-factors(gt) *etc*. and is not relevant to the choice of reflections for refinement. *R*-factors based on *F*^2^ are statistically about twice as large as those based on *F*, and *R*- factors based on ALL data will be even larger.

### Fractional atomic coordinates and isotropic or equivalent isotropic displacement parameters (Å^2^)

**Table:**

	*x*	*y*	*z*	*U*_iso_*/*U*_eq_	
Co1	0.5000	0.5000	0.5000	0.01796 (9)	
P1	0.69129 (7)	0.72084 (7)	0.63065 (4)	0.01948 (10)	
P2	0.80524 (7)	0.75466 (7)	0.40670 (4)	0.01867 (10)	
O1	0.5727 (2)	0.5931 (2)	0.62242 (13)	0.0242 (3)	
O2	0.8322 (3)	0.6463 (2)	0.70245 (15)	0.0287 (3)	
H2	0.8794	0.5361	0.6937	0.043*	
O3	0.5808 (3)	0.9121 (2)	0.66208 (16)	0.0306 (4)	
O4	0.8430 (2)	0.7284 (3)	0.52092 (14)	0.0274 (3)	
O5	0.6535 (2)	0.6611 (2)	0.40048 (13)	0.0245 (3)	
O6	1.0029 (2)	0.6880 (2)	0.33643 (15)	0.0281 (3)	
O7	0.7321 (3)	0.9654 (2)	0.39394 (17)	0.0308 (4)	
H7	0.6270	0.9903	0.3778	0.046*	
O1W	0.7487 (2)	0.2730 (2)	0.49181 (18)	0.0327 (4)	
H1W1	0.748 (6)	0.178 (4)	0.462 (3)	0.049*	
H2W1	0.865 (4)	0.291 (5)	0.477 (3)	0.049*	
N1	0.3681 (3)	0.7717 (3)	0.26576 (15)	0.0258 (3)	
H1A	0.3675	0.8804	0.2865	0.039*	
H1B	0.4577	0.6849	0.2885	0.039*	
H1C	0.2511	0.7508	0.2908	0.039*	
C1	0.4143 (4)	0.7700 (4)	0.15241 (18)	0.0291 (4)	
C2	0.2798 (5)	0.8852 (4)	0.1038 (2)	0.0367 (5)	
C3	0.3238 (7)	0.8820 (6)	−0.0032 (3)	0.0530 (9)	
H3	0.2364	0.9570	−0.0382	0.064*	
C4	0.4949 (8)	0.7695 (7)	−0.0583 (2)	0.0616 (12)	
H4	0.5210	0.7680	−0.1298	0.074*	
C5	0.6260 (6)	0.6602 (6)	−0.0078 (2)	0.0540 (10)	
H5	0.7416	0.5865	−0.0457	0.065*	
C6	0.5902 (5)	0.6569 (4)	0.0994 (2)	0.0378 (6)	
C7	0.0963 (5)	1.0126 (5)	0.1621 (3)	0.0477 (7)	
H7A	0.0077	0.9431	0.1985	0.072*	
H7B	0.0364	1.0961	0.1147	0.072*	
H7C	0.1273	1.0802	0.2102	0.072*	
C8	0.7374 (5)	0.5355 (6)	0.1532 (3)	0.0556 (9)	
H8A	0.8042	0.6090	0.1783	0.083*	
H8B	0.8302	0.4477	0.1057	0.083*	
H8C	0.6715	0.4726	0.2100	0.083*	

### Atomic displacement parameters (Å^2^)

**Table:**

	*U*^11^	*U*^22^	*U*^33^	*U*^12^	*U*^13^	*U*^23^
Co1	0.01549 (15)	0.01757 (15)	0.02352 (17)	−0.00720 (11)	−0.00640 (12)	−0.00003 (12)
P1	0.0174 (2)	0.01835 (19)	0.0248 (2)	−0.00437 (15)	−0.00820 (16)	−0.00249 (16)
P2	0.01319 (18)	0.01767 (19)	0.0260 (2)	−0.00567 (14)	−0.00438 (15)	−0.00018 (16)
O1	0.0256 (7)	0.0265 (7)	0.0252 (7)	−0.0133 (5)	−0.0071 (5)	−0.0016 (5)
O2	0.0286 (7)	0.0271 (7)	0.0330 (8)	−0.0007 (6)	−0.0171 (6)	−0.0051 (6)
O3	0.0286 (7)	0.0206 (6)	0.0450 (10)	−0.0003 (6)	−0.0174 (7)	−0.0082 (6)
O4	0.0206 (6)	0.0389 (8)	0.0276 (7)	−0.0137 (6)	−0.0087 (5)	0.0010 (6)
O5	0.0236 (6)	0.0279 (7)	0.0279 (7)	−0.0154 (5)	−0.0083 (5)	0.0032 (5)
O6	0.0176 (6)	0.0248 (7)	0.0369 (9)	−0.0044 (5)	0.0010 (6)	0.0012 (6)
O7	0.0235 (7)	0.0185 (6)	0.0539 (11)	−0.0040 (5)	−0.0166 (7)	−0.0014 (6)
O1W	0.0191 (6)	0.0259 (7)	0.0549 (12)	−0.0031 (6)	−0.0118 (7)	−0.0099 (7)
N1	0.0265 (8)	0.0297 (8)	0.0232 (8)	−0.0104 (7)	−0.0062 (6)	0.0003 (6)
C1	0.0362 (11)	0.0345 (10)	0.0227 (9)	−0.0204 (9)	−0.0061 (8)	0.0002 (8)
C2	0.0475 (14)	0.0433 (13)	0.0310 (11)	−0.0272 (12)	−0.0166 (10)	0.0089 (10)
C3	0.078 (3)	0.068 (2)	0.0340 (14)	−0.045 (2)	−0.0286 (16)	0.0146 (14)
C4	0.099 (3)	0.080 (3)	0.0228 (12)	−0.057 (3)	−0.0082 (16)	0.0011 (14)
C5	0.070 (2)	0.065 (2)	0.0286 (13)	−0.0376 (19)	0.0110 (14)	−0.0132 (13)
C6	0.0413 (13)	0.0433 (13)	0.0295 (11)	−0.0205 (11)	0.0030 (10)	−0.0084 (10)
C7	0.0455 (16)	0.0484 (17)	0.0554 (19)	−0.0149 (13)	−0.0249 (15)	0.0147 (14)
C8	0.0376 (15)	0.061 (2)	0.057 (2)	−0.0017 (15)	0.0009 (14)	−0.0131 (17)

### Geometric parameters (Å, º)

**Table:**

Co1—O5^i^	2.0645 (18)	N1—H1B	0.8900
Co1—O5	2.0645 (18)	N1—H1C	0.8900
Co1—O1	2.0744 (17)	C1—C2	1.386 (4)
Co1—O1^i^	2.0744 (17)	C1—C6	1.396 (4)
Co1—O1W^i^	2.130 (2)	C2—C3	1.391 (4)
Co1—O1W	2.130 (2)	C2—C7	1.500 (5)
P1—O1	1.4905 (17)	C3—C4	1.383 (7)
P1—O3	1.495 (2)	C3—H3	0.9300
P1—O2	1.5505 (18)	C4—C5	1.369 (7)
P1—O4	1.6151 (19)	C4—H4	0.9300
P2—O5	1.4910 (17)	C5—C6	1.395 (4)
P2—O6	1.4971 (18)	C5—H5	0.9300
P2—O7	1.5529 (19)	C6—C8	1.509 (6)
P2—O4	1.6110 (19)	C7—H7A	0.9600
O2—H2	0.8200	C7—H7B	0.9600
O7—H7	0.8200	C7—H7C	0.9600
O1W—H1W1	0.856 (18)	C8—H8A	0.9600
O1W—H2W1	0.867 (18)	C8—H8B	0.9600
N1—C1	1.473 (3)	C8—H8C	0.9600
N1—H1A	0.8900		
			
O5^i^—Co1—O5	180.0	C1—N1—H1B	109.5
O5^i^—Co1—O1	90.34 (7)	H1A—N1—H1B	109.5
O5—Co1—O1	89.66 (7)	C1—N1—H1C	109.5
O5^i^—Co1—O1^i^	89.66 (7)	H1A—N1—H1C	109.5
O5—Co1—O1^i^	90.34 (7)	H1B—N1—H1C	109.5
O1—Co1—O1^i^	180.0	C2—C1—C6	123.4 (3)
O5^i^—Co1—O1W^i^	93.83 (8)	C2—C1—N1	117.4 (2)
O5—Co1—O1W^i^	86.17 (8)	C6—C1—N1	119.2 (2)
O1—Co1—O1W^i^	92.18 (8)	C1—C2—C3	117.1 (3)
O1^i^—Co1—O1W^i^	87.82 (8)	C1—C2—C7	122.5 (3)
O5^i^—Co1—O1W	86.17 (8)	C3—C2—C7	120.4 (3)
O5—Co1—O1W	93.83 (8)	C4—C3—C2	121.2 (4)
O1—Co1—O1W	87.82 (8)	C4—C3—H3	119.4
O1^i^—Co1—O1W	92.18 (8)	C2—C3—H3	119.4
O1W^i^—Co1—O1W	180.0	C5—C4—C3	120.0 (3)
O1—P1—O3	116.44 (11)	C5—C4—H4	120.0
O1—P1—O2	112.85 (11)	C3—C4—H4	120.0
O3—P1—O2	107.98 (11)	C4—C5—C6	121.5 (4)
O1—P1—O4	108.32 (10)	C4—C5—H5	119.2
O3—P1—O4	108.67 (12)	C6—C5—H5	119.2
O2—P1—O4	101.45 (10)	C5—C6—C1	116.8 (3)
O5—P2—O6	116.77 (11)	C5—C6—C8	120.5 (3)
O5—P2—O7	111.33 (10)	C1—C6—C8	122.8 (3)
O6—P2—O7	109.58 (11)	C2—C7—H7A	109.5
O5—P2—O4	109.55 (9)	C2—C7—H7B	109.5
O6—P2—O4	104.85 (10)	H7A—C7—H7B	109.5
O7—P2—O4	103.77 (11)	C2—C7—H7C	109.5
P1—O1—Co1	133.68 (10)	H7A—C7—H7C	109.5
P1—O2—H2	109.5	H7B—C7—H7C	109.5
P2—O4—P1	129.66 (11)	C6—C8—H8A	109.5
P2—O5—Co1	134.76 (11)	C6—C8—H8B	109.5
P2—O7—H7	109.5	H8A—C8—H8B	109.5
Co1—O1W—H1W1	116 (3)	C6—C8—H8C	109.5
Co1—O1W—H2W1	120 (3)	H8A—C8—H8C	109.5
H1W1—O1W—H2W1	111 (3)	H8B—C8—H8C	109.5
C1—N1—H1A	109.5		
			
O3—P1—O1—Co1	99.39 (16)	O1—Co1—O5—P2	17.64 (15)
O2—P1—O1—Co1	−134.89 (14)	O1^i^—Co1—O5—P2	−162.36 (15)
O4—P1—O1—Co1	−23.38 (17)	O1W^i^—Co1—O5—P2	109.84 (16)
O5^i^—Co1—O1—P1	178.85 (15)	O1W—Co1—O5—P2	−70.16 (16)
O5—Co1—O1—P1	−1.15 (15)	C6—C1—C2—C3	1.6 (4)
O1^i^—Co1—O1—P1	10 (100)	N1—C1—C2—C3	−179.6 (2)
O1W^i^—Co1—O1—P1	−87.30 (15)	C6—C1—C2—C7	−176.8 (3)
O1W—Co1—O1—P1	92.70 (15)	N1—C1—C2—C7	2.0 (4)
O5—P2—O4—P1	−35.28 (19)	C1—C2—C3—C4	−0.4 (5)
O6—P2—O4—P1	−161.35 (15)	C7—C2—C3—C4	178.1 (3)
O7—P2—O4—P1	83.69 (17)	C2—C3—C4—C5	−0.9 (6)
O1—P1—O4—P2	47.67 (18)	C3—C4—C5—C6	1.0 (6)
O3—P1—O4—P2	−79.70 (17)	C4—C5—C6—C1	0.1 (5)
O2—P1—O4—P2	166.66 (15)	C4—C5—C6—C8	−179.8 (4)
O6—P2—O5—Co1	114.85 (16)	C2—C1—C6—C5	−1.5 (4)
O7—P2—O5—Co1	−118.29 (16)	N1—C1—C6—C5	179.7 (2)
O4—P2—O5—Co1	−4.09 (18)	C2—C1—C6—C8	178.5 (3)
O5^i^—Co1—O5—P2	−89 (100)	N1—C1—C6—C8	−0.3 (4)

Symmetry code: (i) −*x*+1, −*y*+1, −*z*+1.

### Hydrogen-bond geometry (Å, º)

**Table:**

*D*—H···*A*	*D*—H	H···*A*	*D*···*A*	*D*—H···*A*
O2—H2···O6^ii^	0.82	1.72	2.532 (3)	174
O7—H7···O3^iii^	0.82	1.70	2.505 (3)	167
O1*W*—H2*W*1···O4^ii^	0.87 (2)	2.11 (2)	2.947 (3)	161 (4)
O1*W*—H1*W*1···O7^iv^	0.86 (2)	1.96 (2)	2.813 (3)	177 (4)
N1—H1*C*···O6^v^	0.89	1.94	2.828 (3)	175
N1—H1*A*···O3^iii^	0.89	1.93	2.805 (3)	168
N1—H1*B*···O5	0.89	2.29	3.005 (3)	138
N1—H1*B*···O1^i^	0.89	2.37	3.016 (3)	129
C7—H7*C*···O2^iii^	0.96	2.58	3.497 (5)	160
C7—H7*A*···O6^v^	0.96	2.57	3.343 (4)	138

Symmetry codes: (i) −*x*+1, −*y*+1, −*z*+1; (ii) −*x*+2, −*y*+1, −*z*+1; (iii) −*x*+1, −*y*+2, −*z*+1; (iv) *x*, *y*−1, *z*; (v) *x*−1, *y*, *z*.
